# Ursolic acid reverses liver fibrosis by inhibiting interactive NOX4/ROS and RhoA/ROCK1 signalling pathways

**DOI:** 10.18632/aging.103282

**Published:** 2020-06-03

**Authors:** Sizhe Wan, Fangyun Luo, Chenkai Huang, Cong Liu, Qingtian Luo, Xuan Zhu

**Affiliations:** 1 Department of Gastroenterology, The First Affiliated Hospital of Nanchang University, Nanchang, Jiangxi, China

**Keywords:** ursolic acid, l iver fibrosis, NOX4, RhoA, ROCK1

## Abstract

Liver fibrosis is the reversible deposition of extracellular matrix (ECM) and scar formation after liver damage by various stimuli. The interaction between NOX4/ROS and RhoA/ROCK1 in liver fibrosis is not yet clear. Ursolic acid (UA) is a traditional Chinese medicine with anti-fibrotic effects, but the molecular mechanism underlying these effects is still unclear. We investigated the interaction between NOX4/ROS and RhoA/ROCK1 during liver fibrosis and whether these molecules are targets for the anti-fibrotic effects of UA. First, we confirmed that UA reversed CCl4-induced liver fibrosis. In the NOX4 intervention and RhoA intervention groups, related experimental analyses confirmed the decrease in CCl4-induced liver fibrosis. Next, we determined that the expression of NOX4 and RhoA/ROCK1 was decreased in UA-treated liver fibrotic mice. Furthermore, RhoA/ROCK1 expression was decreased in the NOX4 intervention group, but there was no significant change in the expression of NOX4 in the RhoA intervention group. Finally, we found that liver fibrotic mice showed a decline in their microbiota diversity and abundance, a change in their microbiota composition, and a reduction in the number of potential beneficial bacteria. However, in UA-treated liver fibrotic mice, the microbiota dysbiosis was ameliorated. In conclusion, the NOX4/ROS and RhoA/ROCK1 signalling pathways are closely linked to the development of liver fibrosis. UA can reverse liver fibrosis by inhibiting the NOX4/ROS and RhoA/ROCK1 signalling pathways, which may interact with each other.

## INTRODUCTION

Hepatic fibrosis is the net accumulation of extracellular matrix (ECM) resulting from chronic liver injury of any aetiology, including viral infection, alcohol consumption, non-alcoholic fatty liver disease, cholestasis, and autoimmune liver disease [1, 2]. ECM accumulation then induces fibrous connective tissue hyperplasia, replacing the space in which normal hepatocyte regeneration occurs [3]. Sustained hepatic fibrosis can lead to cirrhosis, which contributes to more than 1 million deaths per year worldwide [4, 5]; despite this high mortality rate, there is currently no approved anti-fibrotic treatment. Hepatic stellate cells (HSCs) are activated by injury and release ECM, the deposition of which is a central event of liver fibrosis [6]. Once chronic liver disease progresses to end-stage liver disease, there are no effective treatments other than liver transplantation, which is limited by donor shortages, high costs, and immune rejection. Therefore, the reversibility of liver fibrosis has been the subject of extensive research.

NADPH oxidase (NOX) is a multi-subunit transmembrane enzyme complex composed of seven members: NOX1, NOX2, NOX3, NOX4, NOX5 and the two dual oxidases Duox1 and Doux2. The subunits of NOX are slightly different and participate in liver fibrosis by generating reactive oxygen species (ROS) to regulate HSC signal transduction [7]. NOX4, an important subtype of the NOX family, has been shown to induce the conversion of HSCs to myofibroblasts (MFBs) by releasing ROS, which is closely related to liver fibrosis. This function indicates that NOX4/ROS play an important role in the development of liver fibrosis.

To date, more than 20 Rho family members have been discovered. The RhoA subfamily is a group of small GTPase proteins that belong to the Rho protein family, which in turn belongs to the Ras superfamily; when activated, these small proteins act as molecular switches to regulate the cyclical transformation between the activated GTP-binding state and the inactivated GDP-binding state. RhoA binds to multiple target proteins, including epidermal growth factor receptor (EGFR) and Rho-associated coiled-coil-forming protein kinase (ROCK), and regulates cytoskeletal dynamics and gene transcription [8], thereby regulating the adhesion, movement, and contraction of HSCs and participating in the development of liver fibrosis [9].

Studies have shown that Rho GTPases, especially Rac1, can regulate the activation of NOX1 and NOX2 [10], indicating a link between the Rho GTPase family and the NOX family. However, there is controversy about the relationship between NOX4/ROS and RhoA/ROCK. Recent studies have indicated that in pulmonary fibrosis, NOX4/ROS can activate the RhoA/ROCK signalling pathway, promote lung fibroblast migration and collagen synthesis, and enhance pulmonary fibrosis development [11]. However, the role of NOX4/ROS in kidney fibrosis is different from that in pulmonary fibrosis. RhoA/ROCK are upstream signalling molecules of NOX4/ROS. Activation of the RhoA/ROCK signalling pathway can upregulate NOX4/ROS expression, promote renal muscle fibroblast differentiation, and aggravate renal fibrosis [12]. The mechanism of interaction between NOX4/ROS and RhoA/ROCK in liver fibrosis has not been determined, although both NOX4/ROS and RhoA/ROCK are involved in regulating HSC activation in association with the progression of fibrotic disease [11, 13].

Ursolic acid (UA), a traditional Chinese medicine, is a natural pentacyclic triterpenoid compound derived from Chinese medicine plants and has been reported to have anti-inflammatory, anti-fibrotic, and liver protective properties [14, 15]. Although previous studies have confirmed that UA could reverse liver fibrosis by inhibiting the activation of HSCs and promoting apoptosis [16], the specific mechanism of action involved is not clear. This study mainly explored the potential mechanism of the anti-fibrotic properties of UA, providing powerful experimental support for the future clinical application of UA in the treatment of patients with liver fibrosis.

## RESULTS

### UA reverses liver damage and fibrosis in liver fibrotic mice

**Figure 1:**
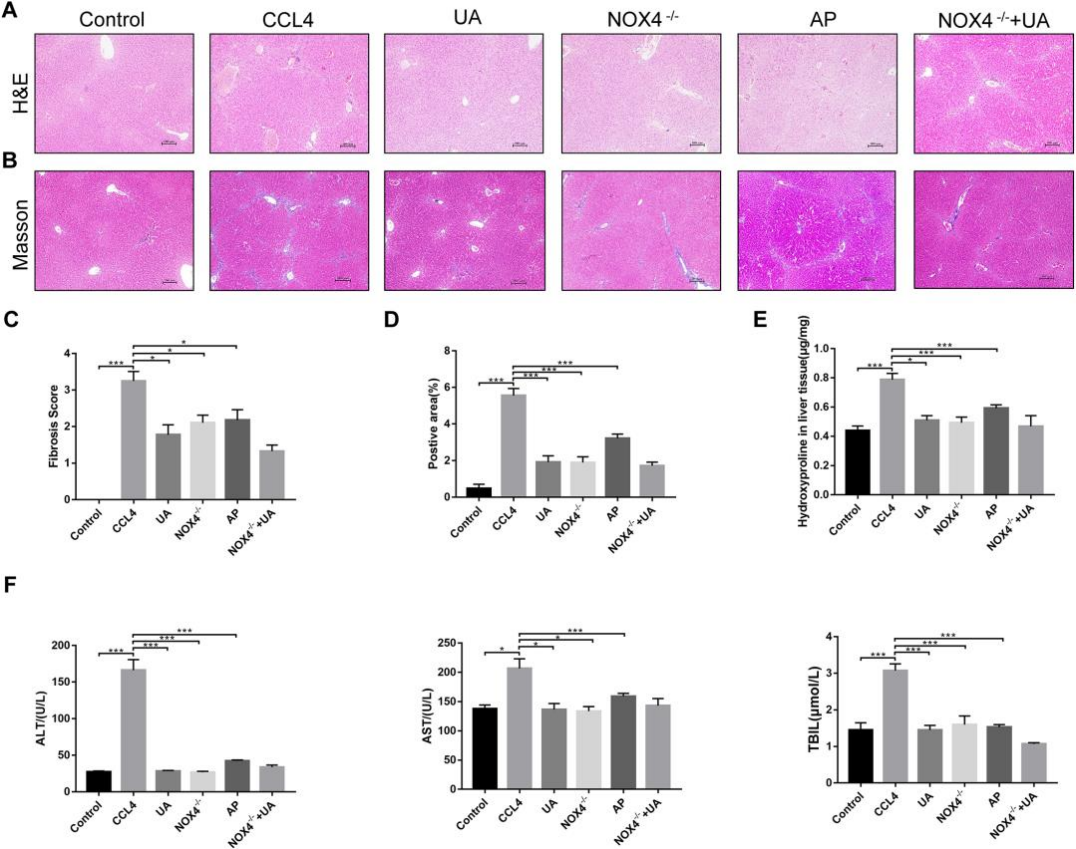
**The effect of UA on CCl_4_-induced liver injury and fibrosis is related to NOX4.** (**A**) HE staining (100× magnification). (**B**) Masson’s trichrome staining (100× magnification). (**C**–**D**) Morphometrical analysis of the fibrotic score and fibrotic area. (**E**) Detection of the hydroxyproline content in the liver tissue by colorimetry. (**F**) Liver function indices in mouse sera. Data represent the mean ± SD for each group. *P < 0.05 and ***P < 0.001.

First, we used HE and Masson’s trichrome staining of mouse liver sections to determine the effects of UA on liver damage, fibrous septum formation and collagen deposition in mice with liver fibrosis (Figure 1A–1B). After CCl_4_-induced mice were treated with UA, the effects of CCl_4_ on liver hepatic lobule structure, collagen deposition and fibrous connective tissue hyperplasia were significantly reversed and were accompanied by decreased inflammatory cell infiltration (P<0.05) (Figure 1C–1D). Furthermore, as an important component of collagen deposition during liver fibrosis, the hydroxyproline content in the liver tissue was also detected (Figure 1E). Next, we assessed the levels of ALT, AST, and TBIL in the mouse serum to determine liver function (Figure 1F). Compared to those in the control group, the serum levels of ALT, AST and TBIL in the CCl_4_ group mice were significantly increased. However, this increase was inhibited in UA-treated fibrotic mice. These results indicate that UA can reverse liver damage and fibrosis to a certain extent to protect the liver.

**Figure 2:**
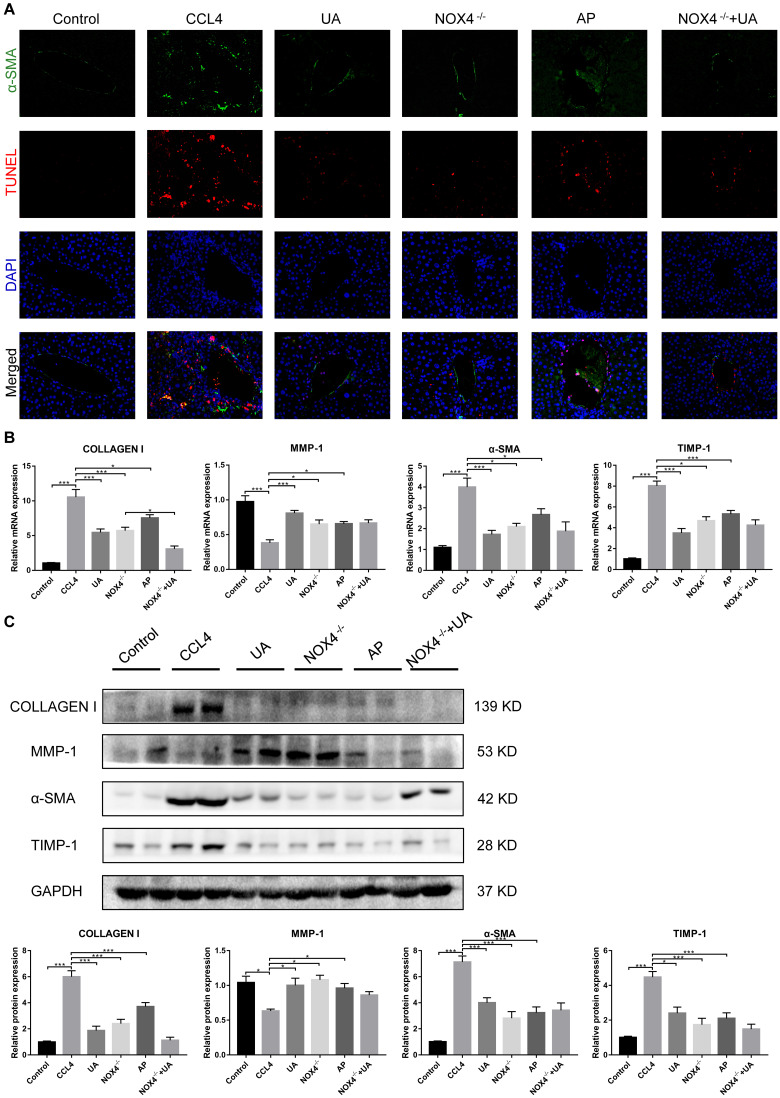
**The effect of UA on CCl_4_-induced liver fibrosis-related indicators is related to NOX4.** (**A**) Dual immunofluorescence staining of liver sections from mice in the control, CCl_4_ and UA groups for nuclei (DAPI, blue), aHSCs (α-SMA, green), and apoptosis (TUNEL, red), and the merged images are shown. (**B**) Hepatic mRNA levels of collagen I, MMP-1, α-SMA, and TIMP-1 were measured by qRT-PCR. (**C**) Collagen I, MMP-1, α-SMA, and TIMP-1 protein expression was detected by a western blot. Data represent the mean ± SD of each group. *P < 0.05 and ***P < 0.001.

HSC activation is well established as the central driver of hepatic fibrosis in experimental and human liver injury, and HSC activation releases a large amount of ECM, which is deposited in the interstitial space of the liver, eventually leading to liver fibrosis [19]. As shown by both α-SMA staining and the TUNEL assay, the expression of α-SMA, a biomarker of activated HSCs [20], in the liver tissue of the CCl_4_ group was significantly increased compared to that in the control group. Apoptosis in hepatocytes in the CCl_4_ group also increased, but there was a significant decrease in apoptosis in the UA group (Figure 2A). This indicates that UA can inhibit the apoptosis of hepatocytes and the activation of HSCs, thereby inhibiting the progression of liver fibrosis.

Liver fibrosis is often accompanied by changes in the expression of fibrosis-related factors. At the mRNA and protein levels, the expression levels of type I collagen and TIMP-1 in the CCl_4_ group were significantly elevated compared with those in the control group. After UA treatment, this increase in the expression of type I collagen and TIMP-1 decreased, and the expression of MMP-1, an anti-fibrotic factor that promotes the degradation of extracellular matrix [21], was elevated (Figure 2B–2C), demonstrating that UV exerts a reversal effect on liver fibrosis.

### UA relieves liver fibrosis by inhibiting the NOX4/ROS signalling pathway

**Figure 3:**
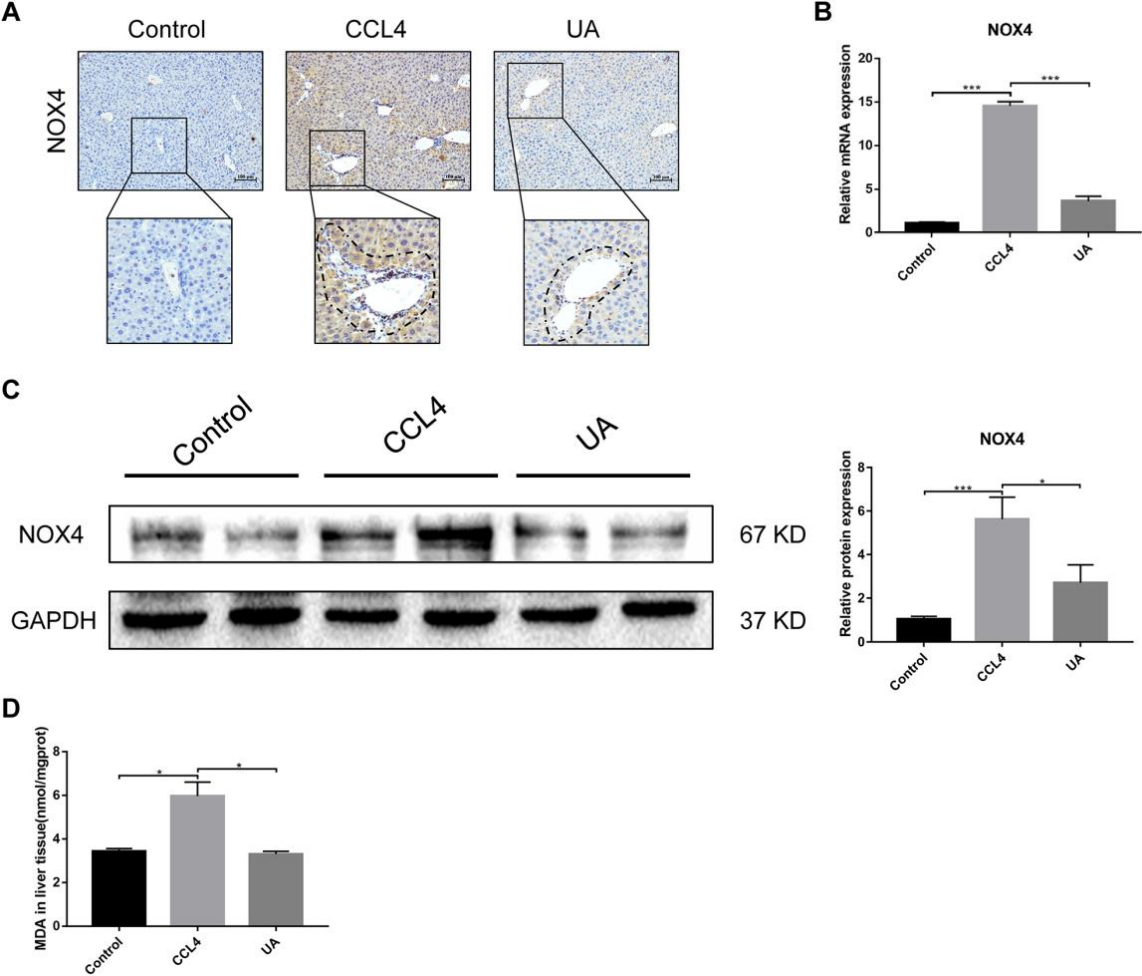
**Effect of UA on the expression of NOX4 in mice with liver fibrosis.** (**A**) The effect of UA on NOX4 expression was determined by using IHC. (**B**) Hepatic mRNA levels of NOX4 were measured by qRT-PCR. (**C**) Hepatic protein levels of NOX4 were detected by a western blot. (**D**) Detection of MDA content in the liver by a commercial kit. Data represent the mean ± SD of each group. *P < 0.05 and ***P < 0.001.

NOX4 mediates the signal transduction of major pro-hepatic fibrosis factors, such as TGF-β, leading to HSC activation and hepatocyte apoptosis and thus plays an important role in liver fibrosis [22]. Our previous studies confirmed that NOX4 and ROS are upregulated in fibrotic liver tissue and that Rac1 expression is upregulated in activated HSCs. In CCl_4_-induced liver fibrotic mice, the NOX4 expression level MDA content that assessed the accumulation of ROS and oxidative stress in the liver were significantly increased (Figure 3A–3D). To further validate the importance of NOX4 in liver fibrosis, we generated NOX4^-/-^ liver fibrotic mice and injected liver fibrotic mice with the NOX4 inhibitor AP (Supplementary Figure 1). Compared with the mice in the CCl_4_ group, the mice in the NOX4^-/-^ and AP groups had a smaller fibrous septum and less collagen deposition, which were accompanied by decreased inflammatory cell infiltration (Figure 1A–1E). The levels of serum markers suggested that liver function damage was reduced in liver fibrotic mice after NOX4 inhibition (Figure 1F). The results of other experiments, such as α-SMA staining, TUNEL assay, MDA content, and liver fibrosis-related factor expression analyses, were similar (Figure 2A–2C), indicating that the inhibition of NOX4 can improve liver fibrosis, confirming that NOX4 is an important profibrotic factor in the progression of liver fibrosis.

To confirm that NOX4 is a target for UA intervention in fibrosis, we first verified the effect of UA on NOX4 expression in vivo by IHC (Figure 3A). The expression of NOX4 in the UA group was lower than that in the CCl_4_ group. Changes in the mRNA expression of NOX4 also confirmed this hypothesis (Figure 3B). The mRNA expression level of NOX4 was significantly lower in the UA group than in the CCl_4_ group (P<0.01). Changes in NOX4 expression at the protein level were also similar to those at the mRNA level (Figure 3C). Moreover, the MDA content in the UA group was lower than that in the CCl_4_ group (Figure 3D), indicating the inhibitory effect of UA on NOX4/ROS. Next, we explored whether NOX4 is a target for the anti-fibrotic effects of UA. In the NOX4^-/-^+UA group, changes to the fibrous septum, collagen deposition and inflammatory infiltration and serum indices were not more decreased than those in the NOX4^-/-^ group, although these changes were still less pronounced than those in the CCl_4_ group (Figure 1A–1F). Furthermore, compared to those in the NOX4^-/-^ group, the number and area of α-SMA- and TUNEL-positive cells in the NOX4^-/-^+UA group were not significantly changed. The changes in factors related to liver fibrosis were similar (Figure 2A–2C), suggesting that UA reverses liver fibrosis by inhibiting the NOX4/ROS pathway.

### RhoA/ROCK1 is another target of the anti-fibrotic effect of UA

**Figure 4:**
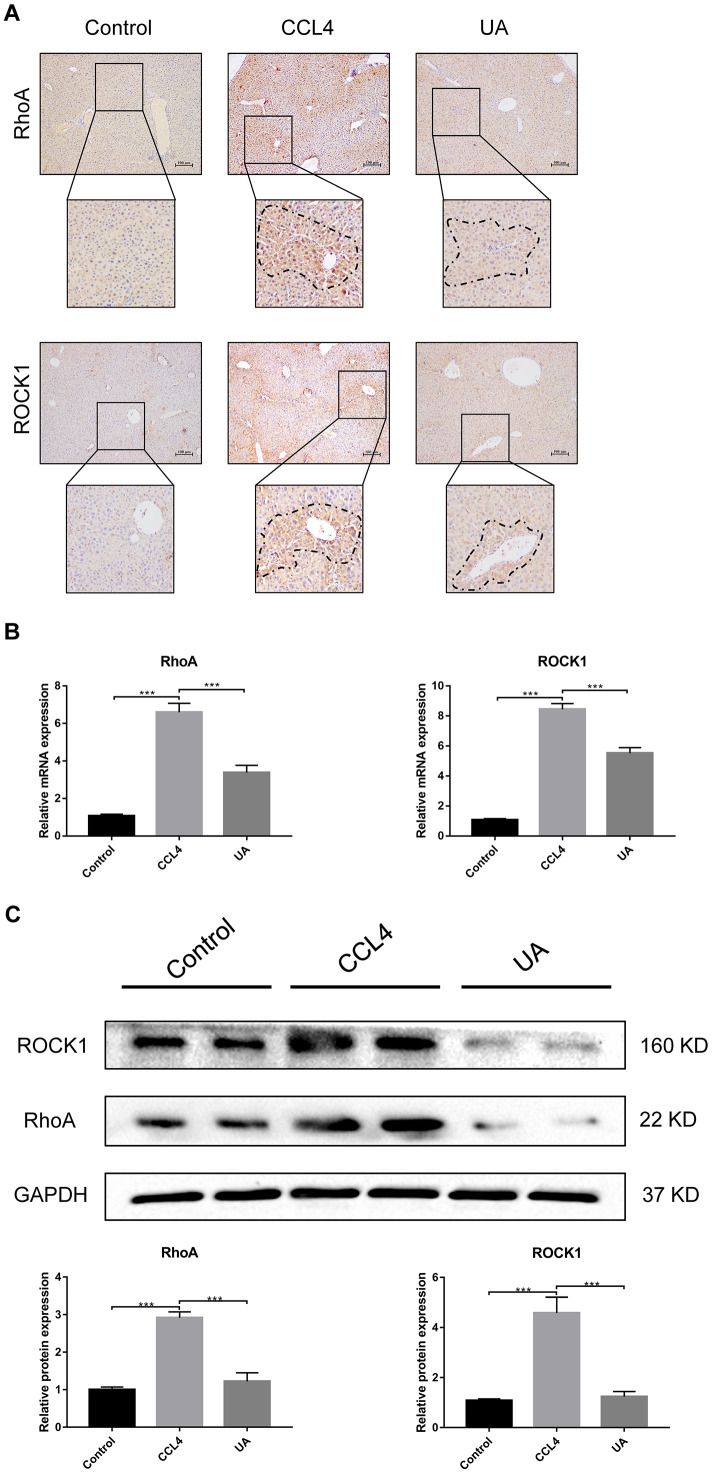
**Effect of UA on the expression of RhoA/ROCK1 in liver fibrotic mice.** (**A**) The effect of UA on RhoA/ROCK1 expression was determined by using IHC. (**B**) Hepatic mRNA levels of RhoA/ROCK1 were measured by qRT-PCR. (**C**) Hepatic protein levels of RhoA/ ROCK1 were detected by a western blot. Data represent the mean ± SD of each group. *P < 0.05 and ***P < 0.001.

**Figure 5:**
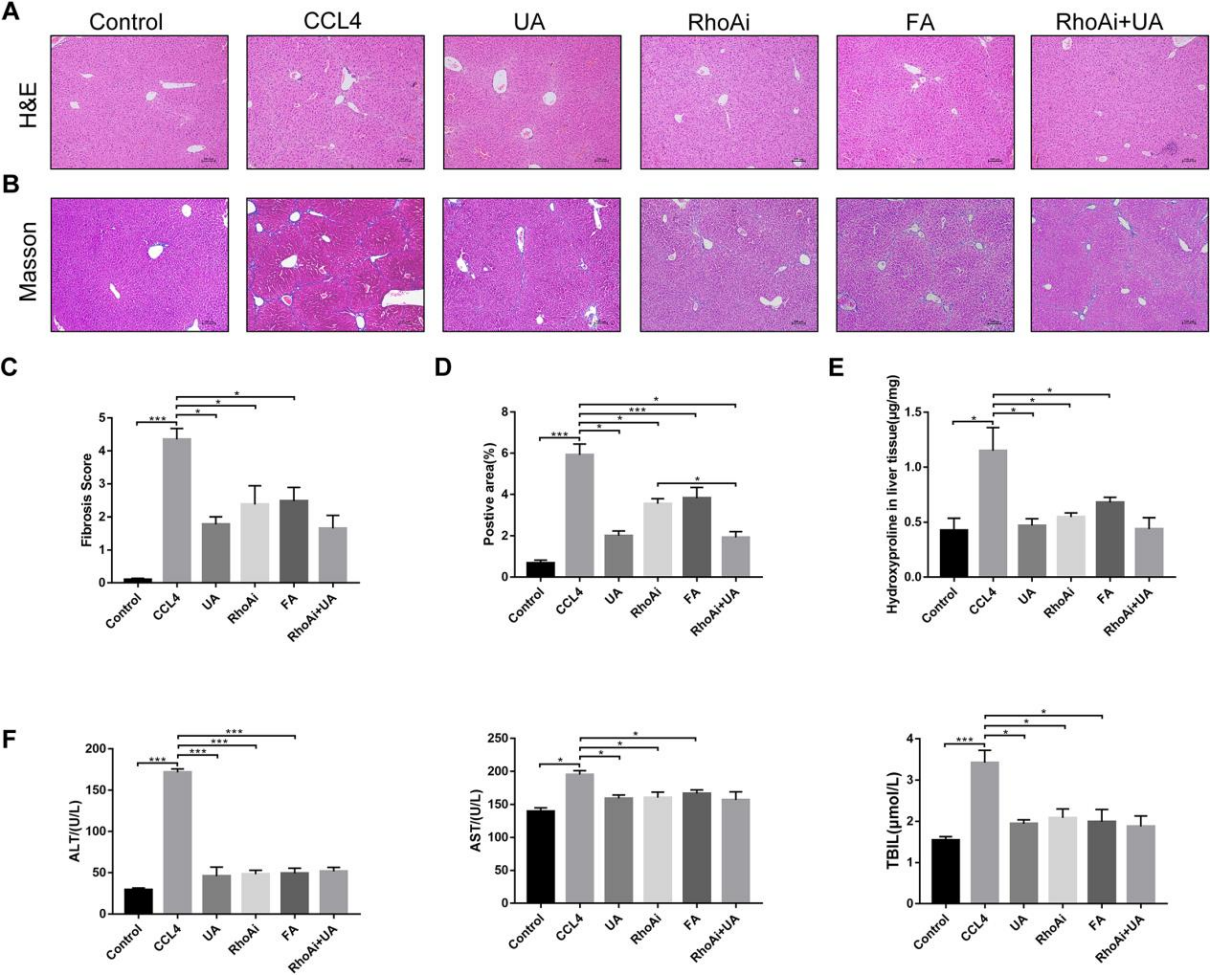
**The effect of UA on CCl_4_-induced liver injury and fibrosis is related to RhoA.** (**A**) HE staining (100× magnification). (**B**) Masson’s trichrome staining (100× magnification). (**C**–**D**) Morphometrical analysis of the fibrotic score and fibrotic area. (**E**) Detection of the hydroxyproline content in liver tissue by colorimetry. (**F**) Liver function indices in mouse sera. Data represent the mean ± SD of each group. *P < 0.05 and ***P < 0.001.

**Figure 6:**
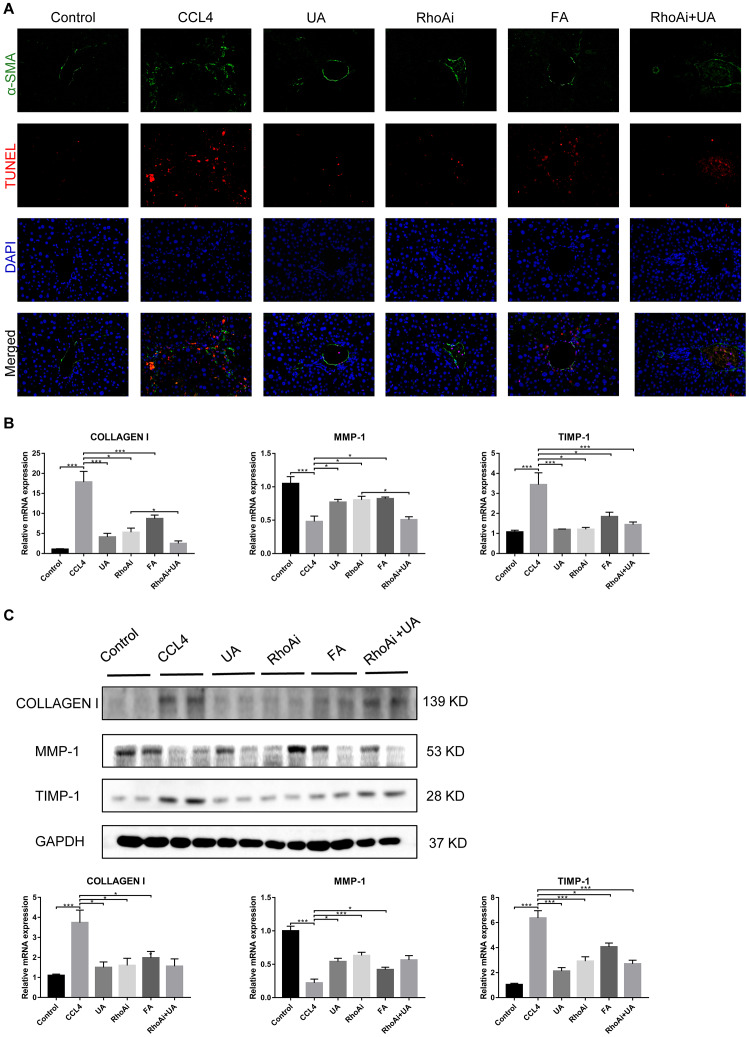
**The effect of UA on CCl_4_-induced liver fibrosis-related indicators is related to RhoA.** (**A**) Dual immunofluorescence staining of liver sections from mice in the control, CCl_4_, and UA groups stained for nuclei (DAPI, blue), aHSCs (α-SMA, green), and apoptosis (TUNEL, red), and the merged images are shown. (**B**) Hepatic mRNA levels of collagen I, MMP-1, α-SMA, and TIMP-1 were measured by qRT-PCR. (**C**) Collagen I, MMP-1, and TIMP-1 protein expression was detected by a western blot. Data represent the mean ± SD of each group. *P < 0.05 and ***P < 0.001.

RhoA has been shown to regulate HSC activation, migration, adhesion, contraction, proliferation and apoptosis [9, 23, 24]. Compared with their expression in the control group, RhoA and ROCK1 expression in the CCl_4_ group was not significantly altered (Figure 4A–4C). To examine the effects of RhoA on liver fibrosis in vivo, we constructed RhoA-inhibited liver fibrotic mice by injecting the mice with AAV and the RhoA inhibitor fasudil (Supplementary Figure 2). Compared with mice in the CCl_4_ group, liver fibrotic mice in the RhoAi and FA groups exhibited a smaller fibrous septum, less collagen deposition and fewer infiltrated inflammatory cells (Figure 5A–5D). Correspondingly, the hydroxyproline content was lower in RhoA-inhibited mice with liver fibrosis than in control mice (Figure 5E). We next tested the levels of ALT, AST, and TBIL in mouse serum. The liver function in liver fibrotic mice in the RhoAi and FA groups exhibited significant recovery, unlike that in the CCl_4_ group (Figure 5F). The results of dual immunofluorescence and the expression of liver fibrosis-related factors also show the importance of RhoA in liver fibrosis (Figure 6A–6C).

We assessed whether UA reverses liver fibrosis by inhibiting RhoA-related signalling pathways. First, to verify the effect of UA on the expression of RhoA and ROCK1, the downstream target of RhoA, we conducted IHC analyses (Figure 4A). The expression of RhoA/ROCK1 in the UA group was lower than that in the CCl_4_ group. Changes in the mRNA and protein levels of RhoA and ROCK1 were similar. In liver fibrotic mice, UA significantly reduced this increase in the expression of RhoA/ROCK1 in the liver (P<0.05) (Figure 4B–4C), indicating that UA can interfere with the expression of RhoA/ROCK. Next, we explored whether the anti-fibrotic effect of UA is related to its inhibition of RhoA/ROCK. Changes to the fibrous septum, collagen deposition and inflammatory infiltration in the RhoAi+UA group were not significant compared with those in the RhoAi group, although they were still less than those in the CCl_4_ group (Figure 5A–5E). The levels of serum indicators also recovered to a certain degree compared with those in the RhoAi treatment group (Figure 5F). Compared to those in the RhoAi group, the number and area of α-SMA- and TUNEL-positive cells were decreased in the RhoAi+UA group (Figure 6A). The expression of liver fibrosis-associated factors also decreased in UA-treated liver fibrotic mice following RhoA inhibition (Figure 6B–6C). These results indicate that UA exerts anti-fibrotic effects by inhibiting the RhoA/ROCK1 signalling pathway.

### NOX4/ROS is the upstream signalling pathway of RhoA/ROCK1 in liver fibrosis

**Figure 7:**
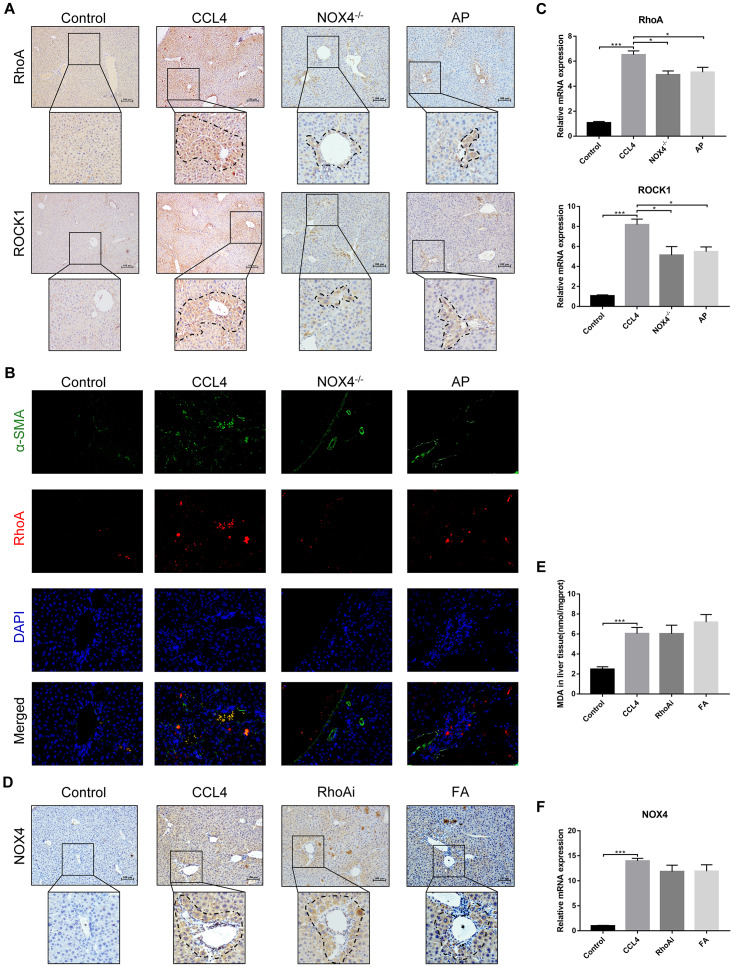
**Interaction between NOX4/ROS and RhoA/ROCK1 in liver fibrotic mice.** (**A**) In liver fibrotic mice in which NOX4 expression was inhibited, RhoA/ROCK1 expression was detected by IHC. (**B**) Dual immunofluorescence staining of liver sections from mice in the control, CCl_4_, NOX4^-/-^ and AP groups stained for nuclei (DAPI, blue), aHSCs (α-SMA, green), and RhoA (red), and the merged images are shown. (**C**) Hepatic mRNA levels of RhoA/ROCK1 were measured by qRT-PCR. (**D**) In liver fibrotic mice in which RhoA expression was inhibited, NOX4 expression was detected by IHC. (**E**) Detection of the MDA content in the liver by a commercial kit. (**F**) Hepatic mRNA levels of NOX4 were measured by qRT-PCR. Data represent the mean ± SD of each group. *P < 0.05 and ***P < 0.001.

The above results confirm that NOX4/ROS and RhoA/ROCK are two signalling pathways on which UA exerts its anti-fibrotic effects. We then sought to clarify the possible interaction mechanism between these two signalling pathways. First, we investigated the expression of RhoA/ROCK1 in NOX4-inhibited liver fibrotic mice. IHC analysis showed that the expression of RhoA/ROCK1 in the NOX4^-/-^ group was decreased compared with that in the CCl_4_ group (Figure 7A). Similarly, dual immunofluorescence staining for α-SMA and RhoA showed decreased expression of RhoA in NOX4^-/-^ mice (Figure 7B). At the mRNA level, the expression of RhoA/ROCK1 in the NOX4^-/-^ group was significantly decreased compared to that in the CCl_4_ group (P<0.01) (Figure 7C). Next, we determined the expression of NOX4 in RhoA-inhibited liver fibrotic mice. As shown by IHC, the expression of NOX4 in the RhoAi group was not significantly lower than that in the CCl_4_ group (Figure 7D). A slight decrease in the MDA content in the livers of RhoA-inhibited liver fibrotic mice was observed (Figure 7E). Compared to that in the CCl_4_ group, the mRNA level of NOX4 in the RhoAi group was decreased to a certain degree, but this difference was not statistically significant (Figure 7F), indicating that NOX4/ROS is the upstream signalling pathway of RhoA/ROCK1 in liver fibrosis.

### Intestinal improvement in liver fibrotic mice following treatment with UA

**Figure 8:**
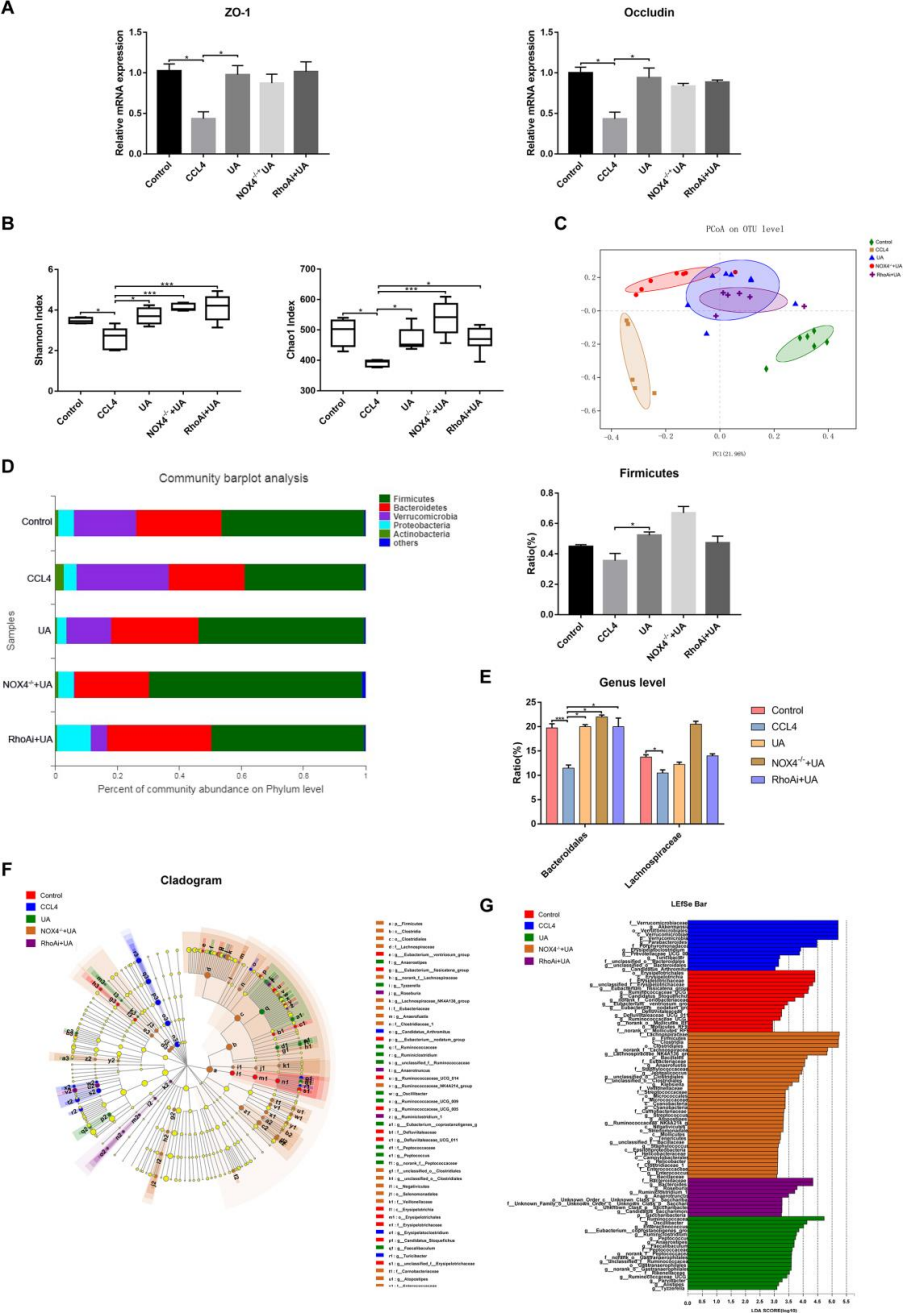
**Effect of UA on intestinal microbiota dysbiosis in mice with CCl_4_-induced liver fibrosis.** (**A**) Ileal mRNA levels of the TJ proteins ZO-1 and occludin were measured by qRT-PCR. (**B**) The alpha diversity of each group was assessed by determining the Shannon index and the Chao1 index. (**C**) PCoA to determine the weighted UniFrac distance of the intestinal microbiota. (**D**) Composition of the intestinal microbiota of each group at the phylum level. (**E**) Composition of the intestinal microbiota of each group at the genus level. (**F**) Linear discriminant analysis effect size (LEfSe) prediction was used to identify the bacteria in each group with the most differential abundance. (**G**) Linear discriminant analysis (LDA) scores showed significant differences in the bacteria in each group. Only the bacteria whose abundance met an LDA threshold value of >2 are shown. Data represent the mean ± SD of each group. *P < 0.05 and ***P < 0.001.

Intestinal damage and destruction of intestinal barrier integrity are often accompanied by liver fibrosis [25–27]. Once the integrity of the intestine is destroyed, harmful factors in the intestine enter the liver through the gut-liver axis, which in turn further aggravates the progression of liver fibrosis [28, 29]. Therefore, we explored improvements to the intestinal barrier induced by UA. As they are key indicators of the integrity of the intestinal barrier, the expression of the tight junction (TJ) proteins ZO-1 and occludin was tested. The expression of ZO-1 and occludin in the ileum of liver fibrotic mice was lower than that in the control group, but their expression increased after UA treatment (P<0.05) (Figure 8A). These results show that UA has a protective effect on the intestinal barrier.

The intestinal microbiota has also received attention as an important indicator of intestinal and systemic status. We explored disorders in the intestinal microbiota of animal models of liver fibrosis and their improvement following treatment with UA. Disorders in the intestinal microbiota in our animal models of liver fibrosis and improvements to the intestinal microbiota following UA treatment were detected by next-generation sequencing. First, we tested some indicators that represent alpha diversity (Figure 8B). The Shannon index and the Chao1 index, which were used to assess microbial abundance, in the CCl_4_ group were significantly decreased compared to those in the control group (P<0.01). After UA treatment, these indices increased (P<0.01). In the NOX4^-/-^+UA and RhoAi+UA groups, this recovery in the Shannon and Chao1 indices was pronounced, but it was not statistically significant. Next, we conducted beta diversity analysis, which was used to compare microbial community compositions and assess differences between microbial communities (Figure 8C). The principal coordinates analysis (PCoA) plot showed a difference in the microbial communities among the control, CCl_4_ and UA groups. Interestingly, there was a slight difference in the microbial communities among the NOX4^-/-^+UA group, the RhoAi+UA group and the UA group. The composition of the intestinal microbiota was also found to change (Figure 8D). At the phylum level, the operational taxonomic units (OTUs) of the beneficial bacteria Firmicutes were lower in the CCl_4_-treated mice than in the control mice. However, after UA treatment, this decrease in the abundance of Firmicutes bacteria was reversed. Firmicutes are beneficial bacteria that protect the body through a variety of mechanisms [30–32]. The abundance of Verrucomicrobia showed the opposite trend. UA also improved microbial abundance at the genus level (P < 0.05). At the genus level, the abundances of Bacteroidales and Lachnospiraceae in the CCl_4_ group were lower than those in the control group. In the UA group, the abundances of both Bacteroidales and Lachnospiraceae increased (Figure 8E). Furthermore, linear discriminant analysis effect size (LEfSe) was used to analyse the microbiota composition (Figure 8F–8G) and revealed the microbiota abundance within each group.

## DISCUSSION

In this study, we show that the traditional Chinese medicine UA can alleviate CCl_4_-induced liver fibrosis. We explored the underlying mechanisms involved in this effect and found that the UA-induced reversal of liver fibrosis may be achieved by inhibiting the NOX4/ROS and RhoA/ROCK signalling pathways, which may interact with each other. The conversion of HSCs to proliferating MFBs, which release ECM that is then deposited, is a central event in the pathogenesis of hepatic fibrosis [33, 34]. NOX has been shown to convert catalytic oxygen molecules to ROS, which in turn regulates the activation of HSCs [35–37]. Furthermore, NOX4 can mediate TGF-β and other signalling pathways and induce the activation of resting HSCs and apoptosis in hepatocytes [38, 39]; NOX4 therefore plays an important role in the progression of liver fibrosis.

In previous experiments, we induced NOX4 activation in HSCs and found that the intracellular ROS levels, proliferation, migration, the degree of cytoskeleton F-actin polymerization and profibrotic factor expression decreased. These results were also confirmed with animal experiments. Injection of NOX4 bioinhibitors into CCl_4_-induced liver fibrosis in mice reversed the progression of liver fibrosis [40]. Here, we used NOX4 knockout mice to better understand the role of NOX4 in the development of liver fibrosis in vivo. Pathological analysis of mouse liver tissue showed that liver fibrosis and inflammatory infiltration in the NOX4^-/-^ group were significantly reduced compared with those in the CCl_4_ group. In NOX4 knockout liver fibrotic mice, the levels of serum indicators that reflect the expression of the ROS indicator MDA and the expression of liver fibrosis-related factors also declined to a certain degree, indicating that the NOX4/ROS signalling pathway plays a role in improving liver fibrosis and that intervention in this pathway can reverse the progression of liver fibrosis.

We also demonstrated in vitro that after induction of RhoA activation in HSCs, cellular proliferation and migration and the degree of cytoskeletal F-actin polymerization increased, and the expression of liver fibrosis-related factors also increased. To demonstrate the role of RhoA in liver fibrosis in vivo, we constructed liver fibrotic mice in which RhoA was inhibited with AAV viruses or inhibitors. Pathological analysis of mouse liver tissue showed that liver fibrosis and inflammatory infiltration in the RhoAi group and FA group were significantly reduced compared with those in the CCl_4_ group. In RhoA-inhibited liver fibrotic mice, the levels of serum indicators and the expression of liver fibrosis-related factors also declined to a certain degree, indicating that the RhoA/ROCK1 signalling pathway plays a role in improving liver fibrosis and that intervention with RhoA can alleviate the progression of liver fibrosis.

The relationship between NOX4/ROS and RhoA/ROCK1, two signalling pathways that play a role in fibrotic diseases, is not clearly defined. In pulmonary fibrosis, NOX4/ROS is an upstream signalling pathway of RhoA/ROCK1 [11]. Interestingly, in renal fibrosis, RhoA/ROCK1 aggravates renal fibrosis by activating the NOX4/ROS signalling pathway [12].

We aimed to determine the relationship between NOX4/ROS and RhoA/ROCK1 in liver fibrosis. We found through previous in vitro experiments that high expression levels of NOX4, but not RhoA, can increase the level of ROS in HSCs. Furthermore, the expression of RhoA was decreased after the inhibition of NOX4 expression. The inhibition of RhoA expression did not affect the expression of NOX4. This result suggests that NOX4 activates HSCs by upregulating the expression of RhoA. In this experiment, we again assessed the relationship between NOX4 and RhoA in an animal model. In the livers of NOX4 knockout liver fibrotic mice, the expression of RhoA and ROCK1 decreased, while in RhoA-inhibited liver fibrotic mice, the expression of NOX4 did not show any obvious change. This suggests that NOX4/ROS may be the upstream signalling pathway of RhoA/ROCK1 in liver fibrosis. In addition, RhoA-related signalling pathways have been shown to be closely linked to human liver cirrhosis and related complications such as portal hypertension [24]. Clarifying the relationship between NOX4/ROS and RhoA/ROCK1 can help elucidate the progression of chronic liver disease.

As a traditional Chinese medicine, UA can inhibit the proliferation of activated HSCs, induce apoptosis, and protect liver cells to exert anti-fibrotic effects [41]. We conducted a preliminary exploration of the target of UA in HSCs and found that UA can inhibit the proliferation and migration of HSCs by inhibiting the NOX4/ROS and RhoA/ROCK1 signalling pathways, and this result was further verified in vivo. In liver fibrotic mice in which NOX4 and RhoA were inhibited, the effect of UA in improving liver fibrosis declined to a certain degree, and the expression of liver fibrosis-related factors was also altered. This suggests that NOX4 and RhoA are two important targets by which UA exerts its anti-fibrotic effects. Unfortunately, limited by objective factors, we have not performed NOX4 and RhoA recovery experiments in animals to verify the relationship between NOX4/ROS and RhoA/ROCK1. More animal models are needed to verify the interaction between NOX4/ROS and RhoA/ROCK1.

Another important finding of this study is the change in intestinal microbiota during UA treatment of liver fibrosis. As adjacent organs, the liver and intestine are closely connected. The two interact and influence each other, and this association is referred to as the "liver-gut axis". Therefore, disorders of the normal metabolic activities of the liver in chronic liver disease cause damage to the intestinal environment through the liver-gut axis [42, 43]. In liver cirrhosis, the intestinal microbiota becomes disordered, which may be due to a reduction in bile acid secretion and changes to the intestinal microbiota composition in cirrhosis, and the excretion of factors in the intestine affects the intestinal microbiota [44]. However, studies on intestinal microbiota changes in liver fibrosis are rare. To this end, we explored changes to the intestinal microbiota during liver fibrosis and its improvement following treatment with UA. In the liver fibrosis animal model, the diversity and abundance of the microbiota were significantly decreased. In terms of the microbiota composition, liver fibrotic mice also have fewer beneficial bacteria than normal mice, indicating a disorder in the intestinal microbiota. Furthermore, the disordered microbiota is likely to stimulate LPS-induced Toll-like receptor (TLR)-related pathway activation through the damaged intestinal barrier, aggravating intrahepatic inflammation and even liver fibrosis [45, 46]. In contrast, when liver fibrotic mice were treated with UA, the decreased diversity and abundance of the intestinal microbiota showed a significant increase, and beneficial bacteria also showed obvious recovery, suggesting that UA improves the intestinal microbiota. Although the current mechanism by which UA improves intestinal microbiota disorders is not clear, this study found that this phenomenon may be related to the inhibition of NOX4 and RhoA by UA-based microbiota changes in the NOX4 intervention and RhoA intervention groups. However, it is unknown whether UA directly affects the intestinal microbiota by inhibiting NOX4 and RhoA or indirectly improves the intestinal microbiota by reducing liver fibrosis. In addition, some researchers have recently attempted to develop characteristic microbiota as a non-invasive biomarker and a microbiota signature for the classification, diagnosis, and treatment of chronic live diseases [47, 48]. Based on the results of this study, we can look for the microbial signature of liver fibrosis to better diagnose liver fibrosis and evaluate the efficacy of UA in clinical. More evidence is required to demonstrate the potential mechanisms of UA in the improvement of the intestinal microbiota, and more sophisticated experiments and models will need to be conducted in the future.

In conclusion, our results indicate that NOX4/ROS and RhoA/ROCK1, which may interact, play an important role in the development of liver fibrosis. Furthermore, UA may reverse liver fibrosis by intervening in these two signalling pathways. The potential mechanism of the anti-fibrotic effects of UA is still being explored. We aimed to clarify the possible molecular targets of UA and provide reasonable experimental evidence for the future use of UA in clinical treatment. These findings provide new insight into UA treatment of liver fibrosis. However, further reasonable in vivo and in vitro experiments are needed to confirm these results. Combined with our previous research results, the results described in this paper provide a reasonable experimental basis for the clinical application of UA.

## MATERIALS AND METHODS

### Experimental animal model and design

The wild-type (WT) C57BL/6 mice used in the experiments were from the Department of Laboratory Animal Science of Nanchang University, and NOX4 knockout C57BL/6 mice were purchased from the Jackson Laboratory (USA). All animals were maintained in a temperature-controlled environment (20–22 °C) with a 12 h light-dark cycle with free access to sterile food and water. Based on widely recognized research, carbon tetrachloride (CCl_4_) was selected to induce liver fibrosis in mice [17, 18]. According to the principle of random allocation, C57BL/6 mice weighing 20 to 30 g were randomly divided into the control group [n = 8, gavage with olive oil (2 ml/kg) twice a week for 8 weeks], CCl_4_ group [n = 8, gavage with CCl_4_ at 2 ml/kg (20% olive oil dilution) twice a week for 8 weeks], and UA group [n = 8, mice that were administered CCl_4_ for 4 weeks received continued CCl_4_ and UA (40 mg/kg/day) gavage for 4 weeks]. NOX4 knockout mice were randomly divided into the NOX4^-/-^ group (n = 8, gavage with CCl_4_ twice a week for 8 weeks) and the NOX4^-/-^ + UA group (n = 8, treatment consistent with that of the UA group). A group of C57BL/6 mice that received adeno-associated virus (AAV) (4 ml/kg) via tail vein injection for 1 week to inhibit RhoA were randomly divided into the RhoAi group (n = 8, gavage with olive oil twice a week for 8 weeks) and the RhoAi + UA group (n = 8, treatment consistent with that of the UA group). We also added 2 inhibitor groups: the apocynin (AP) group [n = 8, mice that were given CCl_4_ by gavage for 4 weeks and CCl_4_ plus AP (40 mg/kg/d) (NOX4 biological inhibitor) by gavage for 4 weeks] and the fasudil (FA) group [n = 8, mice received CCl_4_ by gavage for 4 weeks and CCl_4_ plus FA (10 mg/kg/d) (RhoA biological inhibitor) by gavage for 4 weeks]. The mice were subjected to experimental procedures approved by the Animal Care and Use Committee of Nanchang University. All procedures were performed according to the National Institutes of Health Guide for the Care and Use of Laboratory Animals.

### Measurement of blood indices

Serum samples were collected while sacrificing the mice, and the alanine aminotransferase (ALT), aspartate aminotransferase (AST), and total bilirubin (TBIL) levels were measured by using an automatic biochemical analyser (Department of Clinical Laboratory, First Affiliated Hospital of Nanchang University, China).

### Histological analysis

The liver samples were fixed in 4% neutral-buffered formalin, embedded in paraffin and sectioned. Sections underwent haematoxylin and eosin (HE) staining, Masson’s trichrome staining, immunohistochemistry (IHC), TdT-mediated dUTP nick-end labelling (TUNEL) analysis, and immunofluorescent analysis and were evaluated by microscopy. We randomly selected five visual fields for observation, scored liver fibrosis using METAVIR scoring criteria, and evaluated intestinal mucosal damage using the Chiu scoring method. IHC was used to determine the localization and expression of related proteins. Liver fibrosis was estimated by Masson’s trichrome staining. Specimens were incubated with an appropriate antibody and were observed and photographed by confocal microscopy.

### Hydroxyproline and malondialdehyde measurements

The hydroxyproline and malondialdehyde (MDA) contents of the liver tissues were determined using an assay kit from Nan-jing Jiancheng Bioengineering Institute (Nanjing, China) according to the manufacturer’s instructions.

### Construction and injection of vectors for RhoA inhibition

We constructed an AAV for RhoA inhibition. AAVs carrying short hairpin RNA (shRNA) using CV266 as a vector were injected into the mouse via the tail vein. The final titre of AAV-shRhoA was 3.5 × 10^12^ viral particles/ml.

### Gut microbiota analysis

We used a stool DNA kit (Omega, China) to extract genomic DNA from the faeces collected while sacrificing mice, and then purity was verified by 1% agarose gel electrophoresis (BioFROXX, China). Primers (338F 5'-ACTCCTACGGGAGGCAGCAG-3' and 806R 5'-GGACTACHVGGGTWTCTAAT-3') were used to amplify the bacterial V3-V4 region of the 16S rRNA gene. Pyrosequencing of the PCR products was performed on an Illumina MiSeq Instrument. (Majorbio, China). Analysis of changes in intestinal microbiota by alpha diversity (Shannon and Chao1 index), beta diversity, and community composition were calculated.

### Western blotting analysis

Liver tissue protein was obtained from tissue lysates for western blotting. The protein levels were determined using a BCA assay kit (Tiangen, Beijing, China). Denatured proteins were separated on 10% Tris-glycine polyacrylamide gels by SDS-PAGE and transferred to PVDF membranes. The PVDF membranes were incubated overnight at 4 °C with anti-collagen I (Abcam, Cat. ab138492), anti-MMP1 (Abcam, Cat. ab137332), anti-α -SMA (Abcam, Cat. ab32575), anti-TIMP1 (Abcam, Cat. ab109125), anti-NOX4 (Abcam, Cat. ab109225), anti-RhoA (Abcam, Cat. ab187027), anti-ROCK1 (Abcam, Cat. ab45171), and anti-GAPDH (Abcam, Cat. ab8245). The corresponding membrane-bound antibodies were detected by a hypersensitive chemiluminescence detection reagent.

### Quantitative real-time RT-PCR (qRT-PCR)

For real-time PCR analysis, PCR was performed with a reaction mixture containing cDNA template, primers, and TB Green™ Fast qPCR Mix (TaKaRa) in a Step One Plus Real-Time PCR System (Thermo Fisher Scientific). The relative abundances of the target genes were obtained by comparison against a standard curve.

### Statistical analysis

We used SPSS 23.0 software for data analysis. Image production and output were performed using GraphPad Prism 7.0 software. Each experiment was repeated 3 times to ensure confidence in the results. One-way analysis of variance (ANOVA), Student’s t test, Mann-Whitney rank sum test, or Kruskal-Wallis H test was used to analyse the significant differences between groups. P <0.05 was considered significant.

## Supplementary Material

Supplementary Figures
