# Supplementary material for: Ursolic acid reverses liver fibrosis by inhibiting interactive NOX4/ROS and RhoA/ROCK1 signalling pathways
Source: Aging (Albany NY). 2020 Jun 3;12(11):10614–32. doi: 10.18632/aging.103282 (PMC7346053; doi:10.18632/aging.103282)
Supplement: Supplementary Figures [file aging-12-103282-s001..pdf]

SUPPLEMENTARY FIGURES

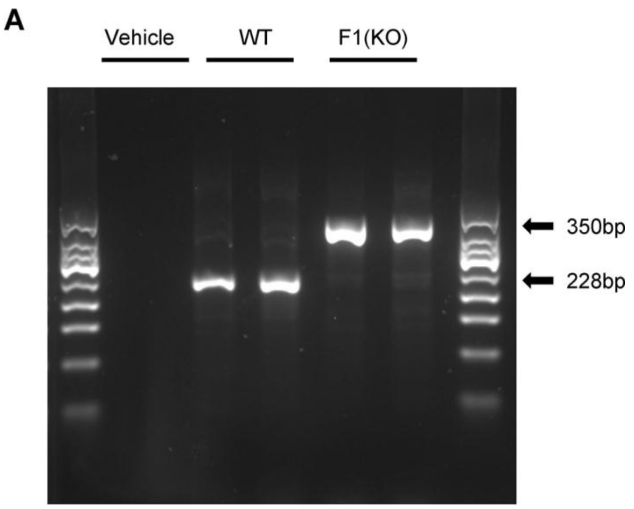

Supplementary Figure 1. NOX4$^{-/-}$ mouse genotype identification. (**A**) NOX4 DNA was validated by agarose gel electrophoresis. WT: wild-type mice; F1: B6.129-NOX4tm1Kkr/J mouse progeny; vehicle: DNA was replaced with water, negative control group.

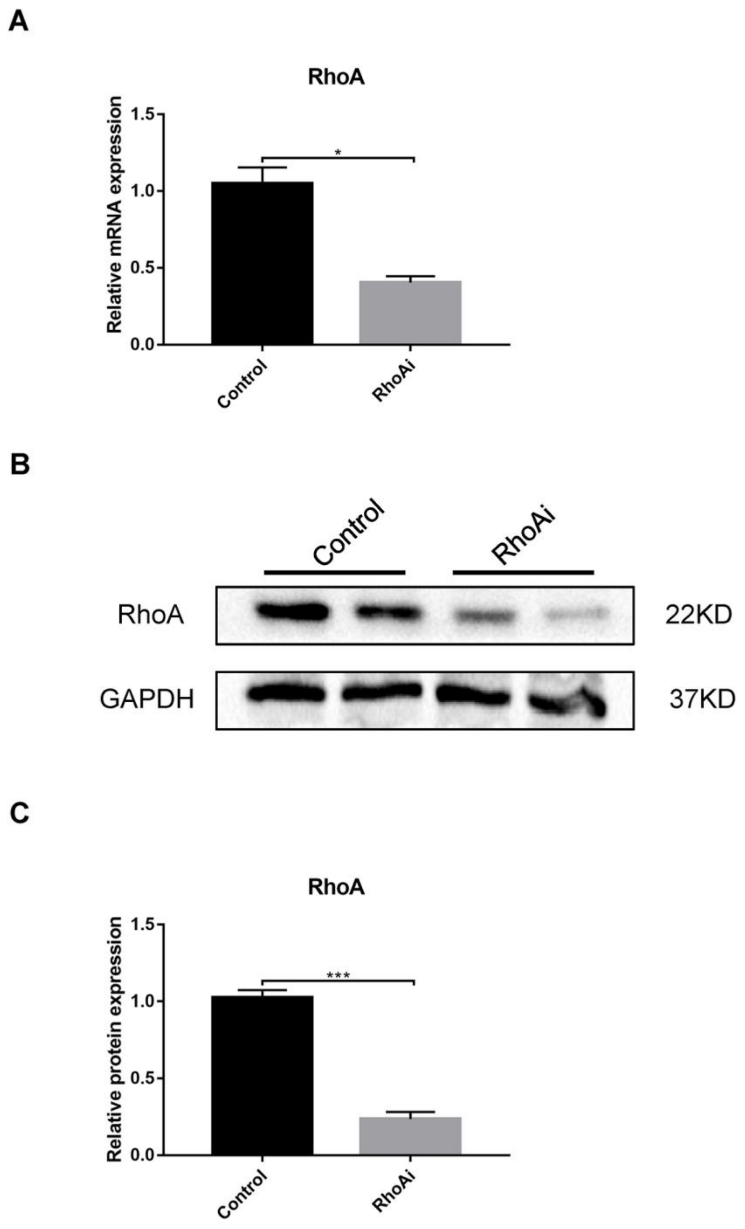

Supplementary Figure 2. Validation of RhoA expression inhibition in the mouse model. (**A**) Hepatic mRNA levels of RhoA were measured by qRT-PCR. (**B**) RhoA protein expression was detected by a western blot. (**C**) Histogram analysis of the levels of RhoA. Data represent the mean ± SD of each group. *P < 0.05 and ***P < 0.001.
